# Workload, Mental Health and Healthcare Among Academics in Australia: A Cross‐Sectional Study

**DOI:** 10.1002/hsr2.72598

**Published:** 2026-06-07

**Authors:** Elisa Zentveld, Huy V. Nguyen, Maxwell Winchester, Stephen L. Edwards, Alan Labas, Fadi Charchar

**Affiliations:** ^1^ Future Regions Research Centre Federation University Mount Helen Victoria Australia; ^2^ Health Innovation and Transformation Centre (HITC) Federation University Mount Helen Victoria Australia; ^3^ Department of Population and Quantitative Health Sciences UMass Chan Medical School Worcester Massachusetts USA; ^4^ Poche Centre for Indigenous Health University of Queensland Brisbane Queensland Australia; ^5^ School of Public Health The University of Queensland Brisbane QLD Australia; ^6^ First Year College Victoria University Melbourne Victoria Australia; ^7^ Department of Marketing Copenhagen Business School Frederiksberg Denmark; ^8^ Centre for Active Living Taylor's University Subang Jaya Selangor Malaysia

**Keywords:** academic, academic staff, anxiety, depression, higher education (HE), mental health, workload

## Abstract

**Background and Aims:**

High stress is associated with adverse mental health outcomes, yet the relationship between stress— measured via workload—and mental health in academics remains underexplored. This study investigated workload, mental health, and healthcare among academics in Australian higher education (HE) institutions.

**Methods:**

In a cross‐sectional study of 626 academics, we collected data on workload, mental health (GAD‐7 and PHQ‐9), and healthcare utilization between February 2022 and August 2022. Associations between excess workload and mental health outcomes were analyzed using both regression and G‐methods.

**Results:**

Academics reported an average annual workload of 3256 h, significantly exceeding the standard 1824 h. Approximately 25% experienced moderate to severe anxiety or depression, substantially higher than estimates in the general population. Mental health service utilization was much higher among academics, yet 30% did not seek help. Each additional 100 h worked beyond 1824 h was associated with 0.10 higher anxiety and depression scores (*β* = 0.10) and 1%–2% increased odds of moderate to severe anxiety and depression. G‐method analyses supported these findings, showing a 2–3 point and a 13%–15% increase in respective scores and likelihood among those working excessive hours.

**Conclusion:**

The findings underscore the significant association between excessive workload and poor mental health among academics in Australian HE institutions. Addressing workload imbalances and improving healthcare services may be associated with better mental health in this population.

## Summary

- Academics work an average of 3256 h annually, exceeding standard limits by 78%.
- One in four academics suffers moderate to severe anxiety or depression in Australia.
- Excessive workload among Australian academics is linked to poor mental health outcomes.
- Mental health service utilization is much higher among academics, yet 30% do not seek help.

## 1 Introduction

The health and wellbeing of Australian academics have emerged as a pressing concern. A large survey conducted between 2020 and 2023 revealed that 43% of Australian university employees reported experiencing extreme tiredness, anxiety, or depression during this period [1]. These issues have worsened annually, reaching the lowest levels in 2023 [1]. While the COVID‐19 pandemic and associated government restrictions have significantly influenced these trends at a macro level, workload and work pressure have been identified as critical micro‐level determinants [2]. A survey by Neser et al. found 66% of Australian university staff reported burnout, academic staff experienced the highest levels of work pressure, and nearly three in five respondents reported conflicts between work and family life [1].

Excessive workload is recognized as a critical risk factor for employee mental health, with several mechanisms linking workload to psychological distress. According to the World Health Organization (WHO), excessive workload is a major source of stress which contributes to poor mental health outcomes [3]. Research by Hassanie et al. indicates that workload impacts mental health both directly and indirectly, with work adaptability mediating this relationship [4]. Heavy workloads are also strongly associated with burnout and exhaustion, common precursors of workplace mental health problems [5]. Despite the importance of workload as a determinant of mental health, research quantifying its impact among academics remains limited and even inconsistent [1, 4].

The relationship between workload and mental health can be conceptualized through established occupational health models. The Job Demand‐Control (JDC) model [6] posits that high job demands combined with low job control increase stress and the risk of adverse health outcomes. Similarly, the Effort‐Reward Imbalance (ERI) model [7] suggests that disproportionate effort relative to rewards leads to psychological strain and burnout. These frameworks provide a theoretical basis for selecting covariates in our analyses, including age, gender, academic level, type of employment, and years of experience, which are considered potential confounders or effect modifiers.

Addressing these research gaps, the present study aims to examine workload, mental health outcomes, and healthcare utilization among Australian academic staff, and to investigate the association between excess workload and mental health outcomes using both regression and G‐methods. Using a regression framework, we sought to provide robust estimates of this association. Regression models are well‐established tools for both prediction and explanation in epidemiological studies [8, 9]. However, recognizing the limitations of our cross‐sectional study design and the potential for residual confounding due to unmeasured covariates, we employed G‐methods to enhance the validity of our findings. These methods, such as inverse probability weighting (IPW) and G‐computation, are designed to balance confounder distributions through re‐weighting exposure [10] or to estimate potential outcomes [11, 12].

Our study extends prior Australian survey‐based research in two key ways. First, existing studies on academic workload have largely relied on subjective perceptions of work pressure or stress [1, 13], without quantifying actual working hours as a measurable exposure. In contrast, we quantify workload using reported annual working hours based on Kenny and Fluck's validated instruments [14, 15, 16, 17, 18, 19], providing a more objective and comparable measure of exposure. Second, while previous studies have used conventional regression approaches, no prior study among Australian academics has applied G‐methods (IPW and G‐computation) to better account for confounding and approximate causal relationships beyond conventional regression approaches [10, 11, 12].

By integrating quantitative workload measures with causal inference methods, our findings aim to provide critical insights into the intersection of workload and wellbeing in higher education (HE). These insights are intended to inform both public health policy and institutional practices, including staffing models, workload allocation frameworks, and mental health screening policies, ultimately promoting healthier work environments in Australian HE.

## 2 Materials and Methods

### 2.1 Study Design and Setting

A cross‐sectional study [20] was conducted across Australian universities between February 2022 and August 2022 using a self‐reported structured questionnaire survey.

### 2.2 Participants and Study Size

Participants who worked as academics—either research only, teaching only, or combining teaching and research—in any HE institution in Australia were eligible for inclusion. To estimate an adequate sample size, a power analysis was conducted using a two‐tailed linear regression model with five variables: workload (exposure) and four covariates. Assuming a power of 0.80, a significance level of 0.05, and a small effect size (Cohen's *f* ^2^ = 0.02) [21], the minimum required sample size was 395 participants. Accounting for potential participant withdrawal (30%) and incomplete responses (15%), the adjusted target sample size was 591 participants to ensure sufficient statistical power and data quality.

### 2.3 Data Sources

Data were collected via an online survey. Participant recruitment began after study protocol approval. Invitations were shared through multiple channels to maximize reach and response rates. Emails were sent to all Australian universities via public contact points, requesting circulation to academic staff. Invitations were also shared through academic networks known to investigators. While some academics may have received multiple invitations, respondents with consent could complete the survey only once. A total of 626 academic staff responded.

### 2.4 Study Variables and Measurement

A structured questionnaire was developed in three sections. The first section collected demographic and academic profile (age, gender, countries of birth, highest academic level, type of employment, number of years working as an academic). The second section was on workload (measured in working hours, see the section below) and mental health (GAD‐7 and PHQ‐9, see the Section 2.4.1). The final section collected mental health services utilization data.

#### 2.4.1 Outcomes (GAD‐7, PHQ‐9)

Two main outcomes, GAD‐7 and PHQ‐9 score, were included for measuring mental health. The GAD‐7 and PHQ‐9 scores were composited from the GAD‐7 and PHQ‐9 scales, respectively. These are among the leading validated and most frequently used depression and anxiety measures and are available in more than 80 translations [22]. GAD‐7 [23, 24] and PHQ‐9 [22, 25] are reliable and valid 7‐item and 9‐item measures of generalized anxiety and depression, respectively, widely used in clinical and research settings. Internal reliability using alpha for GAD‐7 and PHQ‐9 was excellent at 0.92 (equivalent to the standardizing sample) and 0.88. GAD‐7 score was categorized as Minimal: 0–4, Mild: 5–9, moderate: 10–14, severe: 15–21 with highest two deemed “case level” and requiring treatment. PHQ‐9 score was categorized as Minimal: 0–4, Mild: 5–9, Moderate: 10–14, Moderately severe: 15–19, Severe: 20–27, with the latter three case levels. These categories were recoded to establish binary GAD‐7 and binary PHQ‐9 measures, with 1 reflecting moderate or severe anxiety and depression versus 0 minimal or mild. GAD‐7 score and PHQ‐9 score were used as continuous outcomes for linear regression analysis, while binary GAD‐7 and PHQ‐9 were used for logistic regression analysis.

#### 2.4.2 Exposure

Workload questions were developed using the prior research and guidelines by Kenny and Fluck [14, 15, 16, 17, 18, 19] on the different tasks academics undertook in their work. The scoring tools within Qualtrics were then used to calculate annual working hours based on studies by Kenny and Fluck [14, 15, 16, 17, 18, 19]. For standard regression analyses (Table 3), total annual working hours were centered at 1824 h to facilitate interpretation of regression coefficients. The centered variable was calculated as: Workload_centered = Total annual working hours − 1824. Centering allows the intercept in regression models to represent the expected mental health outcome for a standard full‐time workload (1824 h/year). For G‐method analyses, total annual working hours were categorized into a binary variable: 1 for working more than 1824 h per year, and 0 otherwise. This dichotomization improves interpretability and comparability across G‐methods–based estimation. The threshold for both centering and categorization was based on Australia's standard full‐time working hours, typically 38 h per week, adjusted for annual leave, public holidays, sick leave, and other absences, resulting in an approximate total of 1824 h annually [26]. Although G‐methods can accommodate continuous exposures, this dichotomization was a deliberate analytical choice to improve interpretability and facilitate comparisons relative to a meaningful policy‐relevant threshold, rather than a methodological requirement.

### 2.5 Statistical Methods

Categorical and continuous data were summarized as frequencies and percentages, and mean and SDs where appropriate, respectively, to characterize the study sample. Covariates were selected based on theory‐informed reasoning guided by the JDC [6] and ERI frameworks [7] described in the Introduction, which identify age, gender, academic level, type of employment, and years of experience as potential confounders of the workload–mental health relationship. Unadjusted analyses were subsequently conducted to characterize associations between these theory‐selected covariates and mental health outcomes (Supporting Information S1: Tables A1 and A2), and are presented for transparency. The final adjusted regression analyses (Table 3) and G‐methods retained academic level and age group as covariates, as these were identified as the most relevant confounders based on the theoretical framework, corroborated by unadjusted association analyses (Supporting Information S1: Tables A1 and A2), and parsimony given the available sample size.

To complement regression analyses and estimate associations while accounting for confounding. These methods were pre‐specified as part of our analytical strategy to provide additional insight into the workload–mental health relationship under the assumption of no unmeasured confounding, rather than being applied conditionally on the detection of a statistically significant association.

The IPW method estimates the marginal mean potential outcomes as follows:

E[I(A=a)Y/P(A=a|L)=E[Y(a)]]] from which we can estimate the following effect:

ATE=E[Y(1)]−E[Y(0)]=E[I(A=1)Y/P(A=1|L)]−E[I(A=0)Y/P(A=0|L)]

where *A* denotes the binary exposure, *Y* the outcome, *L* the set of measured confounders (age group, gender, academic level, type of employment, and number of academic working years), *I*(·) the indicator function, and *P*(*A *= *a*|*L*) the propensity score.

By re‐weighting each subject's outcome with the inverse probability of receiving the exposure that they experienced, it is possible to create a pseudo‐population in which the confounder distribution is balanced across the entire sample. This allows for the estimation of the average treatment effect (ATE) [10] given several assumptions: the consistency assumption, the correct specification of the propensity score model, and the positivity assumption (i.e., *P(A* = *a*|*L*) > 0 for all *a*). The propensity score, defined as the probability of excess workload (> 1824 h annually) given the measured covariates, was estimated using logistic regression including age group, gender, academic level, type of employment, and number of academic working years.

Covariate balance was assessed using standardized mean differences (SMD) and variance ratios before and after weighting (Supporting Information S1: Table A4). Before weighting, several covariates showed substantial imbalance (SMD up to 0.36). After applying IPW weights, all SMDs were below 0.10, indicating adequate covariate balance across exposure groups. Overlap of propensity scores between exposed and unexposed groups was assessed graphically using a histogram of the estimated propensity scores by exposure group (Supporting Information S1: Figure A1). Adequate overlap was observed across the range of propensity scores, supporting the positivity assumption. The distribution of propensity score was examined to identify extreme values; the mean was 0.72, with a range of 0.18–0.89, indicating no severe violations of positivity. Raw (unstabilized) inverse probability weights were used to estimate the ATE in the entire population. Weight truncation was not applied, as the propensity score distribution showed no extreme values and adequate covariate balance was achieved after weighting (all SMDs < 0.10), indicating that truncation was unnecessary in this instance.

For G‐computation, let *Y* (1) and *Y*(0) represent the two potential outcomes under the exposure and the non‐exposure, respectively, and let *Z* represent exposure status, *Z* = 1 for exposed individuals, and 0 otherwise, and *X* represent the *k* covariates (*X* = *X*_1_,*…, X*_*k*_) measured before exposure. The average treatment effect (ATE) is defined as ATE = *E*[*Y*(1) − *Y*(0)], representing the mean difference between the outcomes of individuals if every subject, regardless of their observed exposure or non‐exposure, had been either exposed or unexposed [11, 12]. G‐computation was adjusted for academic level when estimating GAD‐7 as a continuous outcome, and for age group when estimating binary PHQ‐9, consistent with the unadjusted association analyses (Supporting Information S1: Tables A1 and A2). The propensity score model for IPW included all five theory‐selected covariates (age group, gender, academic level, type of employment, and years of experience) to maximize covariate balance in the pseudo‐population, as IPW relies on the propensity score to balance all measured confounders simultaneously. In contrast, the outcome models for regression and G‐computation retained only the covariates most strongly associated with each specific outcome—academic level for GAD‐7 and age group for binary PHQ‐9—consistent with the unadjusted association analyses (Supporting Information S1: Tables A1 and A2) and the principle of parsimony given the available sample size. By standardizing in this manner, G‐computation can estimate the mean outcome under universal exposure and universal non‐exposure, thereby enabling estimation of the ATE. Given the cross‐sectional nature of the data, these estimates should be interpreted as associations under the assumption of no unmeasured confounding, rather than as causal effects. For IPW analyses, 95% confidence intervals were estimated using robust standard errors (sandwich estimator), while for G‐computation analyses, 95% confidence intervals were derived using the delta method.

**Figure 1:**
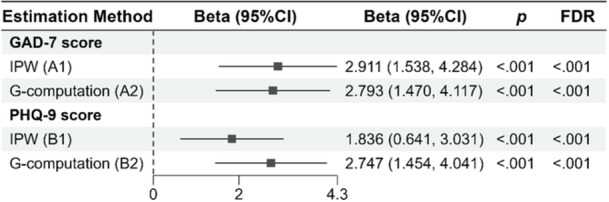
Estimated association between workload and mental health outcomes (measured as continuous scores) using G‐methods. The x‐axis represents the beta coefficient (mean difference in score). A1 and A2: GAD‐7 score; B1 and B2: PHQ‐9 score. A1 and B1: estimated using inverse‐probability weighting (IPW); A2 and B2: estimated using G‐computation. A1 and A2: adjusted for academic level. Exposure: total working hours ≥ 1824 versus < 1824 h per year. Reference line at 0 indicates null association. *N* = 406.

**Figure 2:**
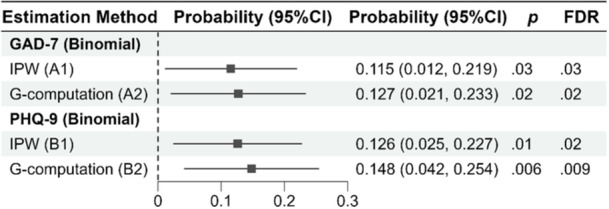
Estimated association between workload and mental health outcomes (measured as binary) using G‐methods. The x‐axis represents the probability difference of moderate‐to‐severe anxiety or depression. A1 and A2: GAD‐7 (binary); B1 and B2: PHQ‐9 (binary). A1 and B1: estimated using inverse‐probability weighting (IPW); A2 and B2: estimated using G‐computation. B1 and B2: adjusted for age group. Exposure: total working hours ≥ 1824 versus < 1824 h per year. Reference line at 0 indicates null association. *N* = 406.

A sensitivity analysis was conducted to assess whether the association of workload with mental health remained consistent. We re‐ran the primary regression analyses after excluding extreme values (outliers) in total adjusted annual working hours, identified using Tukey's method [27]. Specifically, observations with values below Q1 − 1.5 × IQR or above Q3 + 1.5 × IQR were excluded. In total, three respondents were identified as outliers and removed. The regression analyses were then repeated, and the results presented in Supporting Information S1: Table A3 remain consistent. As part of the sensitivity analysis, we further applied both G‐methods, as described above, to both types of outcomes (continuous and binary). The resulting estimates were consistent across methods, indicating the robustness of the findings (Figures 1, 2). The primary analyses, including unadjusted and adjusted regression analyses and G‐methods, were prespecified. The sensitivity analysis applying both G‐methods across continuous and binary outcomes was exploratory.

All hypothesis tests were two‐sided. While a priori significance level of *p* < 0.05 was applied, the Benjamini–Hochberg‐based FDR (false discovery rate) was applied to adjust for multiple testing, meaning that an FDR of < 0.05 was defined as statistical significance [28]. The Benjamini–Hochberg procedure was applied separately within each of the following correction families [1]: the four primary regression analyses [2]; the four sensitivity analyses after outlier exclusion; and [3] the eight G‐method analyses. Correction families were defined based on analytical groupings to reflect the distinct research questions addressed by each set of analyses. All analyses were performed using Stata version 18.5 [29]. Missing data were not imputed. Complete case analysis was performed for each analysis, meaning only participants with complete data on the relevant outcome, exposure, and covariates were included. This accounts for the varying sample sizes observed across analyses (Tables 1, 2, 3; Supporting Information S1: Tables: A1–A4).

### 2.6 Bias

This study considered several potential sources of bias. Selection bias was minimized by distributing survey invitations widely across Australian universities and academic networks. However, because invitations were distributed via public contact points and academic networks, we could not determine the total number of academics who received the invitation, and therefore the response rate could not be calculated. This voluntary response convenience sampling may limit the generalizability of our findings and represents a potential source of selection bias.

Specifically, academics experiencing higher workloads or poorer mental health may have been more motivated to participate, potentially overestimating the prevalence of mental health symptoms and the magnitude of workload–mental health associations in the broader academic population. Conversely, those with the most severe symptoms may have been less able to participate, which could lead to underestimation. As a result, the direction of this bias remains uncertain, and findings should be interpreted with caution when generalizing to all Australian academics. Additionally, the high proportion of Australian‐born participants (99.5%) likely reflects this selection bias, whereby academics reached through institutional mailing lists and domestic academic networks may be disproportionately Australian‐born. Response and recall bias were reduced using validated instruments (GAD‐7 and PHQ‐9), restricting responses to one submission per participant, and excluding extreme workload values. Confounding was addressed through covariate adjustment in regression and G‐methods analyses, and multiple testing was controlled using FDR.

### 2.7 Ethical Approval

Ethics approval was granted by the Federation University Human Research Ethics Committee (HREC No A21‐179). Participants provided informed consent to participate in the survey and agreed to data usage in the research. We did not use artificial intelligence–generated content tools (e.g., ChatGPT or other large language models) to generate any part of this manuscript. Such tools were used only to assist with minor language editing.

## 3 Results

The key characteristics of the sample are outlined in Table 1. The majority of participants were middle‐aged (35–59 years old, 77.2%) and female (64.7%), with nearly all (99.5%) born in Australia. Most were employed full‐time (80.8%), and academics at levels B through D made up 76.7% of the sample. In terms of tenure, a larger proportion of academics had either 1–9 years or 15 or more years of experience, compared to those with 10–14 years.

**Table 1:** Demographic and professional characteristics of the study sample.

Variables	*n* (%)
Age group (*N* = 624)	
< 25–34	67 (10.7%)
35–59	482 (77.2%)
60–65+	75 (12.0%)
Gender (*N* = 624)	
Male	196 (31.4%)
Female	404 (64.7%)
Non‐binary/third gender	9 (1.4%)
Prefer not to say	15 (2.4%)
Countries of birth (*N* = 625)	
Australia	622 (99.5%)
Others	3 (0.5%)
Academic level (*N* = 624)	
Sessional/casual/adjunct academic	50 (8.0%)
Lecturer/level A (assistant professor in US system)	52 (8.3%)
Lecturer/level B (assistant professor in US system)	200 (32.1%)
Senior lecturer/level C (associate professor in US system)	179 (28.7%)
Associate professor/level D (full professor in US system)	99 (15.9%)
Professor/level E (endowed chair in US system)	44 (7.1%)
Type of employment (*N* = 624)	
Full time	504 (80.8%)
Part time	69 (11.1%)
Sessional/casual/adjunct academic	51 (8.2%)
Number of years working as an academic (*N* = 624)	
1–9 years	247 (39.6%)
10–14 years	150 (24.0%)
15 years or more	227 (36.4%)

Table 2 describes workload, mental health, and service use among participants. They worked an average of 3256 h (SD = 2113.84), adjusted for time fraction−—cwell above the standard 1824 h in academia. Teaching (mean = 1370.11, SD = 1106.77) and research (mean = 1354.5, SD = 1095.55) consumed more time than service activities (mean = 503.40, SD = 393.83). Participants reported average anxiety and depression scores near 6, with about one in four experiencing moderate or severe anxiety (25.1%) and depression (24.8%). Overall, 40.6% used at least one mental health service in the past year. Among those with moderate/severe symptoms, 70% sought help (primarily a GP, 36.8%; psychologists, 22.5%; Employee Assistance Programs (EAPs), 14.7%), leaving 30% untreated.

**Table 2:** Descriptives of workload, mental health, and mental health service use among the study sample.

Variables	*n* (%) or mean (SD)
Workload	
Total teaching and HDR hours, mean (SD) (*N* = 405)	1370.11 (1106.77)
Total research hours, mean (SD) (*N* = 335)	1354.53 (1095.55)
Total service hours, mean (SD) (*N* = 406)	503.40 (393.83)
Adjusted total hours (adjusted for time fractions): (*N* = 406)	
Mean (SD)	3256.07 (2113.84)
Median (IQR)	3285.50 (3078)
Mental health (*N* = 626)	
GAD‐7, mean (SD)	5.78 (5.86)
PHQ‐9, mean (SD)	5.73 (5.97)
GAD‐7 (moderate or severe)	157 (25.1%
PHQ‐9 (moderate or severe)	155 (24.8%)
Use of at least one service or more among the entire sample (*N* = 626)	254 (40.6%)
Use of at least one service or more among those with moderate or severe anxiety/depression (*N* = 196)	138 (70.4%)
Telephone counseling service	11 (5.6%)
EAP	29 (14.7%)
GP	72 (36.8%)
Psychiatrist	16 (8.1%)
Mental health nurse	3 (1.6%)
Psychologist	44 (22.5%)
Other counselor	16 (8.5%)
Hospital emergency department	2 (1.3%)
Hospital mental health ward	1 (0.7%)

Abbreviation: IQR, interquartile range

Table 3 displays the results of standard regression analyses for different mental health outcomes, including GAD‐7 and PHQ‐9, both as continuous and binary variables. Each additional hour beyond 1824 annual working hours was associated with a 0.001‐point increase in both the GAD‐7 (anxiety) and PHQ‐9 (depression) scores. The odds of experiencing moderate or higher levels of anxiety and depression (as measured by binary GAD‐7 and PHQ‐9) increased by approximately 0.01%–0.02% for each additional hour of work beyond 1824 h. In practical terms, each additional 100 h of work beyond this threshold corresponded to a 0.10‐point increase in anxiety and depression scores (*β* = 0.10) and a 1%–2% increase in the odds of moderate to severe anxiety or depression. Although the per‐hour effect is modest, the cumulative association is more meaningful in context. The average academic in our sample worked 3256 h annually—approximately 1432 h beyond the standard threshold of 1824 h. At the observed effect size, this excess workload corresponds to an estimated 1.43‐point increase in GAD‐7 and PHQ‐9 scores (1432 × 0.001), and a 14%–29% increase in the odds of moderate to severe anxiety or depression. This cumulative burden, sustained over an academic career, may have substantial implications for long‐term mental health. All associations were statistically significant (*p*'s: < 0.001, < 0.001, 0.01, and 0.005, respectively). Supporting Information S1: Figure A2 further illustrates the positive association between increased workload and higher levels of each of these mental health measures, indicating their consistent trends.

**Table 3:** Adjusted association between workload and mental health as per the standard regression approach.

Main independent variable	Outcome			Outcome		
	Point estimate [95%CI]	*p*	FDR	Point estimate [95%CI]	*p*	FDR
	GAD‐7 score^a^ Beta [95%CI]			PHQ‐9 score^b^ Beta [95%CI]		
Adjusted total hours per year^e^ (*N* = 406)	0.001 [0.0004, 0.0010]	< 0.001	< 0.001	0.001 [0.0002, 0.0008]	< 0.001	< 0.001
	GAD‐7 (binomial)^c^ OR [95%CI]			PHQ‐9 (binomial)^d^ OR [95%CI]		
Adjusted total hours per year^e^ (*N* = 406)	1.0001 [1.00003, 1.0002]	0.01	0.01	1.0002 [1.00005, 1.00025]	0.005	0.007

^a,b^ Linear regression model; a: adjusted for academic level; b: unadjusted.

^c,d^ Logistic regression model; c: unadjusted; d: adjusted for age group.

^e^ Adjusted total hours per year was centered at 1824 h.

Figure 1 demonstrates the results of two robust analytical methods, inverse‐probability weighting (IPW) and G‐computation, to estimate the association of workload with two continuous outcomes: GAD‐7 (anxiety) and PHQ‐9 (depression) scores. Using the IPW method, for those having a total annual working hour > 1824, the GAD‐7 score increased by an estimated 2.91 points (FDR < 0.001). With the G‐computation method, if everyone worked > 1824 h annually, the anxiety score increased by 2.79 points compared to if everyone's annual working hours were below 1824 (FDR < 0.001). For the PHQ‐9 score, the IPW method estimated a 1.84‐point increase for respondents with an annual total working hour > 1824 (FDR < 0.001). The G‐computation method estimated a 2.75‐point increase in PHQ‐9 score if every participant worked > 1824 annually against everyone working below this number (FDR < 0.001). This consistent pattern across both methods strengthens the validity of the observed associations.

Figure 2 indicates the estimated association of workload with two binomial outcomes, binomial GAD‐7 and binomial PHQ‐9. In the IPW analysis, the probability of experiencing moderate or severe anxiety increased by 11.5% for respondents with annual working hours > 1824 (FDR = 0.03). The G‐computation analysis showed the probability of moderate or severe anxiety increased by 12.7% if everyone worked > 1824 h annually compared to if everyone's annual total working hours was below 1824 (FDR = 0.02). For PHQ‐9, the IPW analysis estimated a 12.6% increase in the probability of moderate or severe depression among respondents with an annual total working hour > 1824 (FDR = 0.02). The G‐computation method estimated a 14.8% increase in the probability of moderate or severe depression if every participant worked > 1824 annually against everyone working below this number (FDR = 0.009). Both approaches provided statistically significant estimates, further supporting the validity of these associations.

## 4 Discussion

This study is among the first to comprehensively examine working hours as an indicator of workload and its associations with mental health outcomes and healthcare use among academic staff in Australian HE institutions. The findings point to a substantial workload burden, which is significantly associated with elevated levels of anxiety and depression among academics. Notably, approximately one fourth respondents reported experiencing moderate to severe symptoms of anxiety (25.1%) and depression (24.8%). However, despite this high prevalence, only 40.6% reported using at least one mental health service in the past year. Among those with moderate to severe symptoms, 70% sought professional help, leaving a concerning 30% who remained untreated. These findings underscore the urgent need for institutional and policy‐level interventions to mitigate workload‐related stress and improve access to and uptake of mental health support services.

Academics in our study reported an adjusted annual workload averaging 3256 h, far exceeding the standard 1824 h typical in Australian HE institutions [26]. This elevated workload maybe partly attributable to the cascading impacts of the COVID‐19 pandemic and the subsequent policy responses by HE institutions. As noted by Rahman et al. [2] and Owens et al. [30], the pandemic‐induced decline in international student enrollments prompted cost‐cutting strategies in HE institutions across developed countries, including Australia and the UK. These measures–such as organizational restructuring, staff redundancies, early retirements, and reductions in non‐salary expenditures–have disproportionately increased the workload for remaining staff, adding complexity to their roles and adversely affecting their health and well‐being [2].

Approximately one in four academics experienced moderate to severe anxiety (25.1%) and depression (24.8%), substantially higher than rates observed in the general Australian population, where these conditions affect one in six individuals, respectively [13]. Direct comparisons should be interpreted with caution due to differences in measurement instruments and thresholds used between national surveys and our study (GAD‐7 and PHQ‐9, moderate to severe cut‐offs). Despite this, the prevalence of mental health issues reported in our study was lower than that in other studies on HE staff, such as Rahman et al. [2] (73.1%) and Neser et al. [1] (around 40%). These differences are likely due to variations in measurement tools and thresholds. While the aforementioned studies used the K‐10 scale with varying cut‐offs, our research employed the GAD‐7 and PHQ‐9 scales with thresholds set at moderate to severe. Despite methodological differences, a consistent pattern emerges across studies: mental health issues are common among academic staff in HE settings. Among academics with moderate/severe symptoms, more than 70% sought at least one mental health service, which was considerably higher than the rate among the general Australian adult population aged 16–85 (17.4%) [13], noting that the latter includes individuals across all levels of mental health status. However, the considerable proportion of individuals with moderate to severe symptoms who do not seek mental healthcare (around 30%) suggests the presence of structural, cultural, or psychological barriers that merit further investigation.

Using both standard regression and G‐methods, we quantified the association between excessive workload and mental health outcomes. In standard regression analyses, each additional 100 h worked beyond 1824 annual hours was associated with a 0.10‐point increase in GAD‐7 and PHQ‐9 scores, while the odds of experiencing moderate or severe anxiety or depression rose by 1%–2%. G‐method analyses showed that academics working more than 1824 h annually experienced increases of 2–3 points on the GAD‐7 and PHQ‐9 scales compared to those working fewer hours. Moreover, their probability of experiencing moderate to severe anxiety or depression was 13%–15% higher. These findings highlight excessive workload as a critical modifiable factor associated with the mental health of HE staff.

This study has several limitations. While the use of G‐methods reduces confounding, the cross‐sectional design precludes establishing causality. Reverse causation cannot also be ruled out, as individuals with poorer mental health may perceive their workload as higher or experience reduced productivity, which could in turn influence reported working hours. Additionally, as data were collected via a self‐reported online survey, the study may be susceptible to selection bias, social desirability bias, recall bias in reporting working hours, and common method variance, which could influence the observed associations between workload and mental health outcomes. Specifically, the use of self‐reported measures for both exposure (working hours) and outcomes (mental health symptoms) may inflate observed associations due to shared method variance, and responses may be subject to social desirability bias, whereby participants underreport workload or mental health symptoms. Another limitation is the focus on the direct association between workload and mental health, without exploring alternative pathways such as workload‐induced stress [3], work adaptability [4], or burnout and exhaustion [5]. Investigating these pathways in future research could inform multifaceted interventions, as a single strategy is unlikely to adequately address the complex mental health challenges faced by academic staff. Furthermore, we did not conduct multivariable analyses examining predictors of non‐utilization among symptomatic individuals due to limited sample size and statistical power within this subgroup. Future studies with larger samples should explore these predictors to better understand barriers to mental health service use.

In conclusion, this study provides valuable insights into patterns of workload, mental health, their associations, and healthcare access among academics in Australian HE institutions. The findings underscore the urgent need to prioritize the health and well‐being of this group. In practice, institutions could consider implementing workload monitoring systems, setting upper limits on annual working hours, providing structured mental health support programs, and encouraging staff to access available services. Policies promoting flexible work arrangements, reducing administrative burdens, and recognizing teaching and research contributions may also help mitigate workload‐related stress and improve academic well‐being. Future research should explore reasons for limited access to healthcare and adopt longitudinal designs to establish temporal relationships and explore indirect pathways linking workload to mental health.

## Author Contributions

**Elisa Zentveld:** conceptualization, investigation, methodology, writing – review and editing, project administration, supervision, resources, and funding acquisition. **Huy V. Nguyen:** writing – original draft, methodology, validation, visualization, writing – review and editing, software, formal analysis, and data curation. **Maxwell Winchester:** investigation, writing – review and editing. **Steve L. Edwards:** investigation, writing – review and editing. **Alan Labas:** investigation, writing – review and editing. **Fadi Charchar:** conceptualization, investigation, writing – review and editing.

## Funding

The authors have nothing to report.

## Conflicts of Interest

The authors declare no conflicts of interest.

## Transparency Statement

The lead author, Elisa Zentveld, affirms that this manuscript is an honest, accurate, and transparent account of the study being reported; that no important aspects of the study have been omitted; and that any discrepancies from the study as planned have been explained.

## Supporting information


**Figure A1:** Propensity score overlap between exposure and non‐exposure Blue bars represent the unexposed group (≤1,824 hours/year) and red bars represent the exposed group (>1,824 hours/year). The horizontal axis represents the estimated propensity score of being in the exposed group, and the vertical axis represents the density. Propensity scores were estimated using logistic regression including age group, gender, academic level, type of employment, and number of academic working years.
**Figure A2:** Adjusted association between workload (total working hours) and mental health outcomes as per standard regression approach.
**Table A1:** Unadjusted association between characteristics and mental health (continuous outcomes).
**Table A2:** Unadjusted association between characteristics and mental health (binomial outcomes).
**Table A3:** Adjusted associations between workload and mental health from regression analyses after exclusion of three outliers (Tukey's method).
**Table A4:** Assessment of covariate balance after inverse probability weighting.

## Data Availability

Data is available upon request.
