# Supplementary material for: Workload, Mental Health and Healthcare Among Academics in Australia: A Cross‐Sectional Study
Source: Health Sci Rep. 2026 Jun 7;9(6):e72598. doi: 10.1002/hsr2.72598 (PMC13242695; doi:10.1002/hsr2.72598)
Supplement: Supplementary file 1 — Figure A1: Propensity score overlap between exposure and non‐exposure Blue bars represent the unexposed group (≤1,824 hours/year) and red bars represent the exposed group (>1,824 hours/year). The horizontal axis represents the estimated propensity score of being in the exposed group, and the vertical axis represents the density. Propensity scores were estimated using logistic regression including age group, gender, academic level, type of employment, and number of academic working years. Figure A2: Adjusted association between workload (total working hours) and mental health outcomes as per standard regression approach. Table A1: Unadjusted association between characteristics and mental health (continuous outcomes). Table A2: Unadjusted association between characteristics and mental health (binomial outcomes). Table A3: Adjusted associations between workload and mental health from regression analyses after exclusion of three outliers (Tukey's method). Table A4: Assessment of covariate balance after inverse probability weighting. [file HSR2-9-e72598-s001.docx]

**Appendix**

**Note on covariate selection:** Tables A1 and A2 present unadjusted associations between participant characteristics and mental health outcomes, and are provided for descriptive and transparency purposes only. Covariate selection for adjusted analyses was theory-informed, guided by the Job Demands-Control (JDC) and Effort-Reward Imbalance (ERI) frameworks, as described in the Methods section. Unadjusted analyses served to corroborate theory-informed covariate selection, rather than as the primary criterion for inclusion.

**Table A1. Unadjusted association between characteristics and mental health (continuous outcomes)**

| **Variables** | **GAD-7 score** | | **PHQ-9 score** | |
| --- | --- | --- | --- | --- |
|  | **Beta [95%CI]** | ***p*** | **Beta [95%CI]** | ***p*** |
| **Demographic/academic characteristics** |  |  |  |  |
| Age group (N = 624) |  |  |  |  |
| <25-34 | reference |  | reference |  |
| 35-59 | 0.46 [-1.03, 1.96] | .54 | 0.25 [-1.28, 1.77] | .75 |
| 60-65+ | -0.80 [-2.74, 1.13] | .41 | -1.17 [-3.14, 0.80] | .24 |
| Gender (N = 624) |  |  |  |  |
| Male | reference |  | reference |  |
| Female | 0.30 [-0.71, 1.30] | .56 | 0.30 [-0.72, 1.32] | .57 |
| Non-binary / third gender | -0.06 [-3.99, 3.87] | .98 | 1.10 [-2.90, 5.10] | .59 |
| Prefer not to say | -0.42 [-3.51, 2.67] | .79 | -0.97 [-4.11, 2.18] | .55 |
| Academic Level (N = 624) |  |  |  |  |
| Sessional/Casual/Adjunct | reference |  | reference |  |
| Lecturer/Level A | 0.39 [-1.88, 2.65] | .74 | 0.53 [-1.79, 2.84] | .65 |
| Lecturer/Level B | 0.56 [-1.25, 2.38] | .54 | 0.21 [-1.64, 2.06] | .82 |
| Senior Lecturer/Level C | 1.18 [-0.65, 3.01] | .21 | 0.93 [-0.94, 2.80] | .33 |
| Associate Professor/Level D | 2.11 [0.12, 4.10] | .04 | 1.45 [-0.58, 3.47] | .16 |
| Professor/Level E | -0.96 [-3.32, 1.41] | .43 | -1.65 [-4.06, 0.77] | .18 |
| Type of employment (N = 624) |  |  |  |  |
| Full Time | reference |  | reference |  |
| Part Time | -0.03 [-1.51, 1.44] | .96 | -0.46 [-1.97, 1.05] | .55 |
| Sessional/Casual/Adjunct Academic | -1.05 [-2.74, 0.65] | .23 | -0.96 [-2.68, 0.76] | .27 |
| Number of academic working years (N = 624) |  |  |  |  |
| 1-9 years | reference |  | reference |  |
| 10-14 years | -0.05 [-1.25, 1.14] | .93 | -0.34 [-1.55, 0.88] | .58 |
| 15 years or more | 0.34 [-0.72, 1.40] | .53 | 0.28 [-0.80, 1.36] | .62 |
| **Workload** |  |  |  |  |
| Total teaching & HDR hours (N = 405) | 0.001 [0.0009, 0.0019] | <.001 | 0.001 [0.0007, 0.0018] | <.001 |
| Total research hours (N = 335) | 0.0001 [-0.0004, 0.0007] | .65 | -0.0003 [-0.0009, 0.0002] | .23 |
| Total service hours (N = 406) | 0.0019 [0.0005, 0.0034] | .007 | 0.0018 [0.0003, 0.0033] | .02 |
| Adjusted total hours (N = 406) | 0.0006 [0.0004, 0.0009] | <.001 | 0.0005 [0.0002, 0.0008] | <.001 |

**Table A2. Unadjusted association between characteristics and mental health (binomial outcomes)**

| **Variables** | **Binomial GAD-7** | | **Binomial PHQ-9** | |
| --- | --- | --- | --- | --- |
|  | **OR [95%CI]** | ***p*** | **OR [95%CI]** | ***p*** |
| **Demographic/academic characteristics** |  |  |  |  |
| Age group (N = 624) |  |  |  |  |
| <25-34 | reference |  | reference |  |
| 35-59 | 1.29 [0.70, 2.38] | .41 | 0.71 [0.41, 1.23] | .22 |
| 60-65+ | 0.60 [0.25, 1.41] | .24 | 0.28 [0.12, 0.66] | .004 |
| Gender (N = 624) |  |  |  |  |
| Male | reference |  | reference |  |
| Female | 1.10 [0.74, 1.63] | .64 | 1.01 [0.68, 1.51] | .94 |
| Non-binary / third gender | 1.59 [0.38, 6.59] | .53 | 2.47 [0.64, 9.56] | .19 |
| Prefer not to say | 0.79 [0.21, 2.93] | .73 | 0.77 [0.21, 2.85] | .69 |
| Academic Level (N = 624) |  |  |  |  |
| Sessional/Casual/Adjunct | reference |  | reference |  |
| Lecturer/Level A | 1.33 [0.52, 3.40] | .55 | 1.18 [0.47, 2.96] | .72 |
| Lecturer/Level B | 1.26 [0.59, 2.71] | .55 | 0.94 [0.44, 2.00] | .88 |
| Senior Lecturer/Level C | 1.38 [0.64, 2.99] | .41 | 1.41 [0.67, 2.97] | .36 |
| Associate Professor/Level D | 1.91 [0.85, 4.30] | .12 | 1.62 [0.73, 3.57] | .24 |
| Professor/Level E | 0.89 [0.32, 2.50] | .82 | 0.67 [0.23, 1.91] | .46 |
| Type of employment |  |  |  |  |
| Full Time (N = 624) | reference |  | reference |  |
| Part Time | 1.02 [0.57, 1.80] | .96 | 0.96 [0.54, 1.72] | .89 |
| Sessional/Casual/Adjunct Academic | 0.62 [0.29, 1.30] | .20 | 0.72 [0.35, 1.47] | .36 |
| Number of academic working years  (N = 624) |  |  |  |  |
| 1-9 years | reference |  | reference |  |
| 10-14 years | 0.82 [0.51, 1.33] | .43 | 0.79 [0.49, 1.29] | .35 |
| 15 years or more | 1.07 [0.71, 1.62] | .74 | 1.05 [0.70, 1.58] | .82 |
| **Workload** |  |  |  |  |
| Total teaching & HDR hours (N = 405) | 1.0004 [1.0002, 1.0006] | <.001 | 1.0003 [1.0001, 1.0005] | .005 |
| Total research hours (N = 335) | 0.99 [0.99, 1.0002] | .69 | 1.00 [0.99, 1.0002] | .99 |
| Total service hours (N = 406) | 1.00 [0.99, 1.001] | .08 | 1.00 [0.99, 1.001] | .11 |
| Adjusted total hours (N = 406) | 1.0001 [1.00003, 1.0002] | .01 | 1.0001 [1.00003, 1.0002] | .009 |

**Table A3. Adjusted associations between workload and mental health from regression analyses after exclusion of three outliers (Tukey’s method)**

| **Main independent variable** | **Outcome** | |  | **Outcome** | |  |
| --- | --- | --- | --- | --- | --- | --- |
|  | **Point estimate**  **[95%CI]** | ***p*** | **FDR** | **Point estimate**  **[95%CI]** | ***p*** | **FDR** |
|  | **GAD-7 score ^A^**  **Beta [95%CI]** |  |  | **PHQ-9 score ^B^**  **Beta [95%CI]** |  |  |
| Adjusted total hours per year^E^  (N=403) | 0.001  [0.0004, 0.0010] | <.001 | <.001 | 0.001  [0.0002, 0.0008] | <.001 | <.001 |
|  | **GAD-7 (binomial) ^C^**  **OR [95%CI]** |  |  | **PHQ-9 (binomial) ^D^**  **OR [95%CI]** |  |  |
| Adjusted total hours per year^E^  (N=403) | 1.0001  [1.00004, 1.0002] | .009 | .01 | 1.0001  [1.00003, 1.00025] | .01 | .01 |

A & B: linear regression model; A: adjusted for academic level; B: unadjusted;

C & D: logistic regression model; C: unadjusted; D: adjusted for age group;

E: adjusted total hours per year was centred at 1,824 hours.

**Table A4. Assessment of covariate balance after inverse probability weighting**

| **Covariates** | **Standardised mean differences (SMD)** | | **Variance ratio** | |
| --- | --- | --- | --- | --- |
|  | **Raw** | **Weighted** | **Raw** | **Weighted** |
| Age group |  |  |  |  |
| 35-59 | 0.158646 | -0.03799 | 0.797912 | 1.060182 |
| 60-65+ | 0.011535 | 0.027006 | 1.020963 | 1.06639 |
| Gender |  |  |  |  |
| Female | -0.03851 | -0.04582 | 1.021645 | 1.033799 |
| Non-binary/third gender | -0.14556 | -0.00729 | 0.182918 | 0.916629 |
| Prefer not to say | -0.16885 | 0.021217 | 0.37142 | 1.128134 |
| Academic level |  |  |  |  |
| Lecturer/Level A | -0.33413 | -0.00474 | 0.346776 | 0.983782 |
| Lecturer/Level B | -0.23152 | 0.00674 | 0.851701 | 1.005235 |
| Senior Lecturer/Level C | 0.254037 | -0.03612 | 1.282359 | 0.971611 |
| Associate Professor/Level D | 0.358975 | 0.030146 | 2.040049 | 1.051463 |
| Professor/Level E | 0.048019 | 0.013031 | 1.168089 | 1.044204 |
| Type of employment |  |  |  |  |
| Part Time | -0.34493 | 0.001093 | 0.520822 | 1.002223 |
| Sessional/Casual/Adjunct | -0.17962 | -0.02343 | 0.470043 | 0.905066 |
| Number of academic working years |  |  |  |  |
| 10-14 years | 0.006245 | -0.0072 | 1.002377 | 0.990578 |
| 15 years or more | 0.32051 | 0.034256 | 1.214165 | 1.017348 |


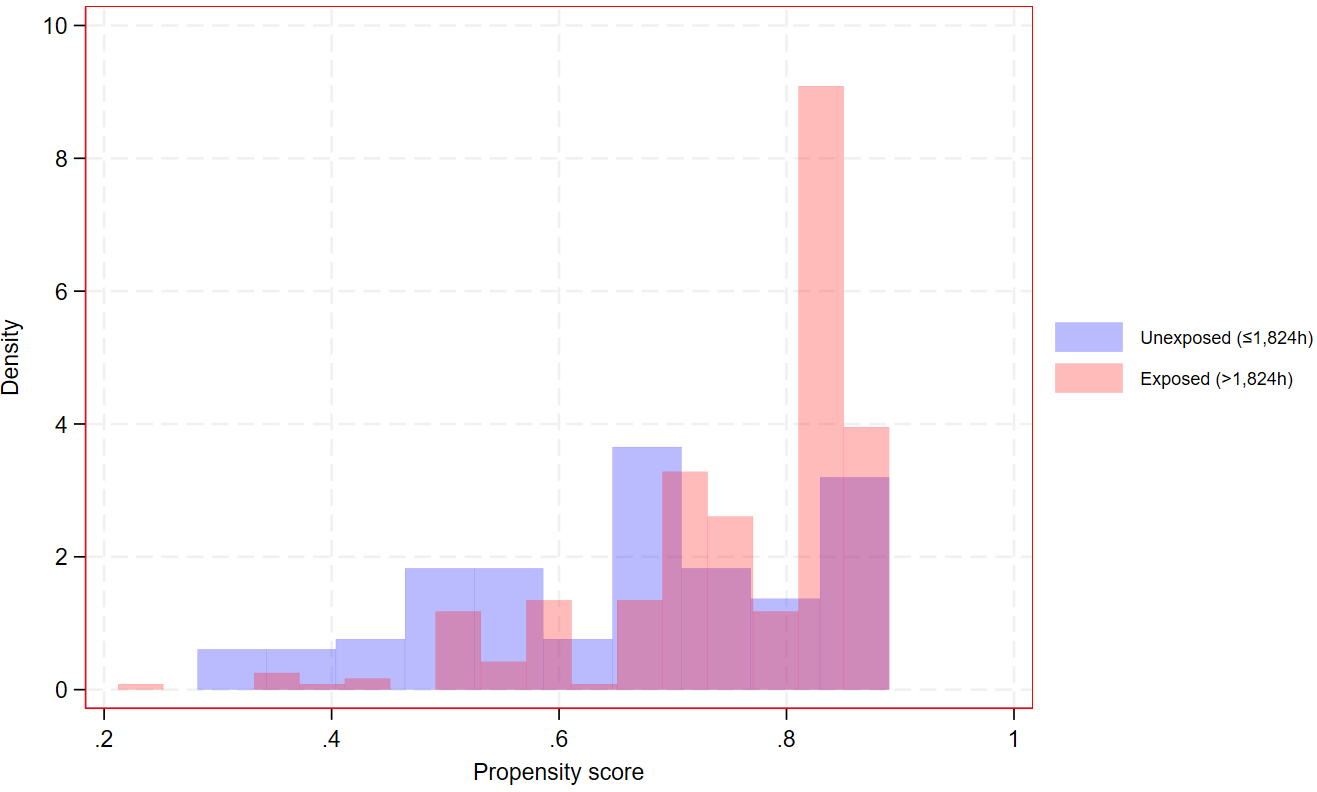


**Figure A1. Propensity score overlap between exposure and non-exposure**

Blue bars represent the unexposed group (≤1,824 hours/year) and red bars represent the exposed group (>1,824 hours/year). The horizontal axis represents the estimated propensity score of being in the exposed group, and the vertical axis represents the density. Propensity scores were estimated using logistic regression including age group, gender, academic level, type of employment, and number of academic working years.


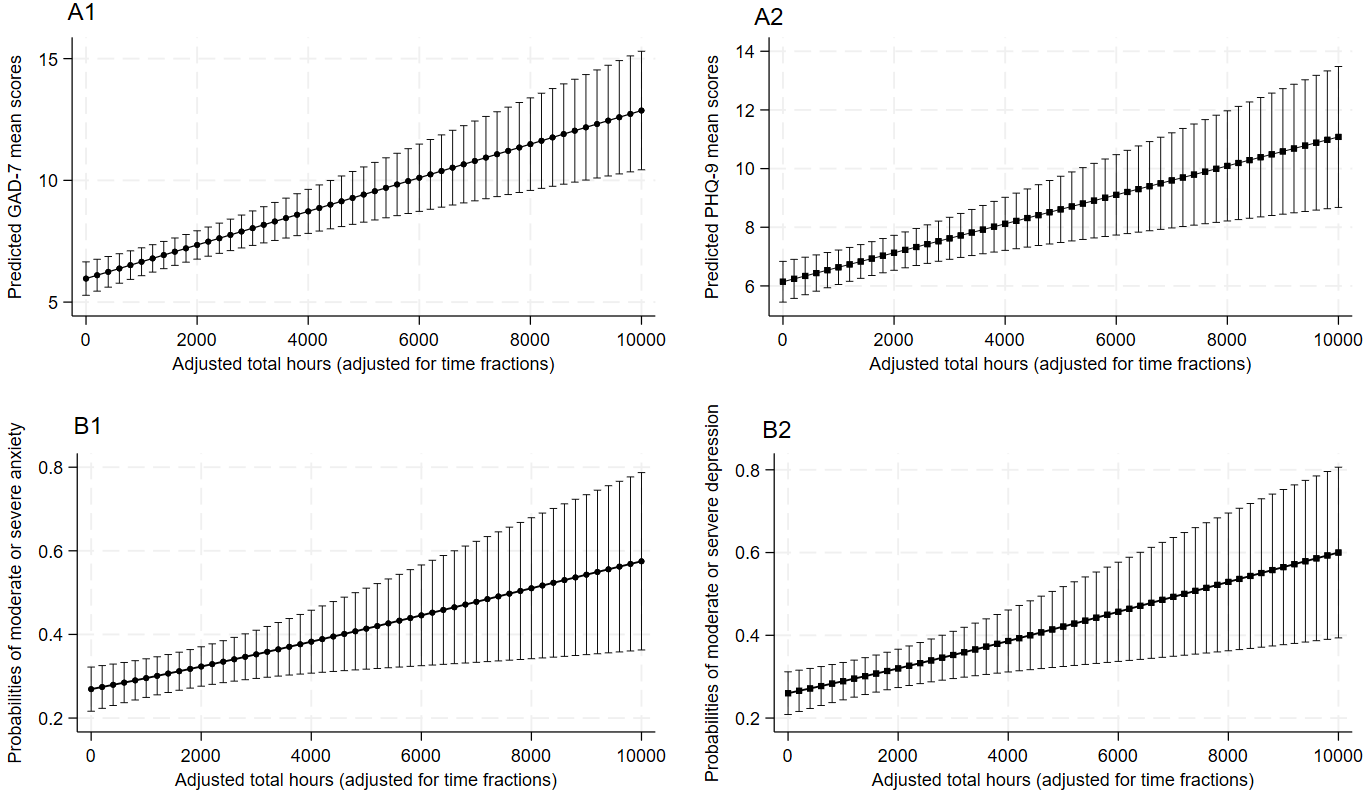


# Figure A2. Adjusted association between workload (total working hours) and mental health outcomes as per standard regression approach
